# Tumor Habitats Based on Multiparametric MRI Distinguish Atypical Glioblastoma From Primary Central Nervous System Lymphoma: Imaging‐Pathologic Correlation

**DOI:** 10.1002/jmri.70080

**Published:** 2025-08-20

**Authors:** Meng‐nan Sun, Hui Wang, Yan‐ying Yang, Xiao‐jun Yu, Hai‐nan Li, Dan‐dan Fu, Dong‐Zhang, Ru‐yu Ai, Xiao‐yu Hua, Li‐chao Wang, Ming‐yao Lai, Chang‐zheng Shi, Lin‐bo Cai

**Affiliations:** ^1^ Medical Imaging Center The First Affiliated Hospital of Jinan University Guangzhou China; ^2^ Department of Oncology Guangdong Sanjiu Brain Hospital Guangzhou China; ^3^ Department of Pathology Guangdong Sanjiu Brain Hospital Guangzhou China; ^4^ Departmentof Radiology and Nuclear Medicine The Fifth Affiliated Hospital of JinanUniversity Heyuan China

**Keywords:** atypical glioblastoma, peritumoral brain edema, primary central nervous system lymphoma, tumor habitat, vasculogenic mimicry

## Abstract

**Background:**

Atypical glioblastoma (GBM) (minimal or no necrosis on MRI) and primary central nervous system lymphoma (PCNSL) are difficult to distinguish on MRI; whether tumor habitat can more accurately distinguish atypical GBM from PCNSL remains uncertain.

**Purpose:**

To evaluate the diagnostic performance with tumor habitats, apparent diffusion coefficient (ADC), and edema index (EI) to distinguish atypical GBM from PCNSL.

**Study Type:**

Retrospective.

**Population:**

One hundred twenty‐five patients (63 male and 62 female) diagnosed with atypical GBM or PCNSL were included.

**Field Strength/Sequence:**

1.5 T and 3.0 T, Axial ADC and T1 contrast‐enhanced spin‐echo inversion recovery sequence (T1‐CE).

**Assessment:**

The tumor habitat was derived using T1‐CE and ADC sequences. Based on this tumor habitat, EI and relative ADC (rADC), we constructed a model.

**Statistical Tests:**

Logistic regression; Akaike Information Criterion; Receiver operating characteristic (ROC) curves, calibration curves, and Decision Curve Analysis.

**Results:**

Three tumor habitats were identified: high‐enhancement cellular habitat (Habitat 1), low‐enhancement cellular habitat (Habitat 2), and nonviable tissue habitat (Habitat 3). The voxel fraction of the three tumor habitats in atypical GBM and PCNSL groups shows statistically significant differences. The EI of patients in the PCNSL group was significantly higher than that of the patients in atypical GBM. A model was established incorporating the parameters Habitat 2, Habitat 3, EI, and rADCmean. The model exhibits excellent discriminative ability in the training set (AUC = 0.851, 95% CI: 0.781–0.921) and validation set (AUC = 0.807, 95% CI: 0.724–0.889). Histopathological evaluation showed that vasculogenic mimicry (VM) levels were significantly higher in the PCNSL group. Multiple linear regression analysis showed a significant correlation between habitat voxel fraction and VM levels.

**Data Conclusion:**

A model built based on tumor habitat, EI, and rADCmean can differentiate atypical GBM from PCNSL preoperatively. The differences in VM levels are one of the pathological mechanisms underlying the variations in tumor habitats between atypical GBM and PCNSL.

**Level of Evidence:**

5.

**Technical Efficacy Stage:**

3.

## Plain Language Summary

This study demonstrates that tumor habitats derived from T1 contrast‐enhanced (TI‐CE) and apparent diffusion coefficient (ADC) sequences can effectively distinguish atypical glioblastoma (GBM) (minimal or no necrosis on MRI) from primary central nervous system lymphoma (PCNSL). CD34‐PAS dual staining assay further confirms that the differences in VM levels are one of the pathological mechanisms for the difference in voxel fraction of tumor habitats between atypical GBM and PCNSL. Despite limitations such as a small sample size and retrospective analysis, this study provides a new method for distinguishing atypical GBM from PCNSL.

**Abbreviations**
AIC: Akaike information criterion
AUC: area under the curve
DCA: decision curve analysis
EI: edema index
GBM: glioblastoma
PCNSL: primary central nervous system lymphoma
PTBE: peritumoral brain edema
rADC: relative ADC
ROC: receiver operating characteristic
VM: vasculogenic mimicry

## 1 Introduction

Glioblastoma (GBM) and primary central nervous system lymphoma (PCNSL) are the two most prevalent types of primary malignant brain tumors [1, 2, 3]. Treatment approaches differ between GBM and PCNSL [4, 5]. Therefore, precise and non‐invasive diagnosis of intracranial PCNSL and GBM holds considerable clinical significance. Atypical GBM [6], which presents minimal or no necrosis on MRI, and PCNSL often exhibit similar imaging characteristics. This overlap in imaging features can create significant challenges for accurate diagnosis. Vasculogenic mimicry (VM) [7], a vessel‐like structure, exists in vascular‐dependent solid tumors and provides a unique source of blood supply. However, the relationship between VM structures and the differing enhancement patterns in GBM and PCNSL remains poorly understood.

Researcher [8] have found that there is tumor heterogeneity between the primary tumor and metastatic lesions, between different metastatic lesions, and even within a single tumor (either primary or metastatic). Subregions assessed by the habitat imaging technique are a manifestation of the tumor microenvironment with adaptations between tumor cells. The use of clustering methods to combine similar voxels for tumor habitat analysis can help to determine the heterogeneity within the tumor [8]. In clinical practice, T1 contrast‐enhanced (T1‐CE) and apparent diffusion coefficient (ADC) are routinely used in the majority of patients with brain tumors; therefore, it is worth exploring whether T1‐CE and ADC imaging‐derived tumor habitats can be used to differentiate between atypical GBM and PCNSL. Peritumoral brain edema (PTBE) is an imaging feature of intracranial malignant tumor [9]. Variations within PTBE could potentially serve as an important factor in differentiating between various malignant brain tumors. To assess the extent of both the tumor and the edema, commonly used methods rely on indirect measurements such as the maximum diameter, width, and two‐dimensional area [10]. However, these approaches are inconsistent, leading to variability. Therefore, a calculation‐based edema index (EI) could offer a more precise way to quantify the relationship between the tumor and surrounding brain edema; the calculation of the EI for each patient is as follows: EI = (voxels tumor + voxels edema)/voxels tumor [9].

The aim of this study was to evaluate the diagnostic value of T1‐CE and ADC imaging‐derived tumor habitats, ADC, and PTBE in the preoperative differentiation of atypical GBM and PCNSL. Additionally, we sought to explore the pathological mechanisms responsible for the differences in tumor habitat between atypical GBM and PCNSL.

## 2 Materials and Methods

### 2.1 Patients Selection

The institutional review committee approved this retrospective study and waived the requirement of informed consent. A total of 681 patients diagnosed pathologically with GBM (*n* = 406) or PCNSL (*n* = 275) were included in the study. Among them, 81 patients were recurrent GBM and 27 were recurrent PCNSL, with 25 and 20 patients, respectively, undergoing stereotactic biopsy. Steroid therapy was administered to 28 GBM patients and 17 PCNSL patients. Additionally, 150 GBM patients and 140 PCNSL patients lacked TICE and/or ADC sequences. Poor image quality was observed in 5 GBM patients and 7 PCNSL patients.

**FIGURE 1:**
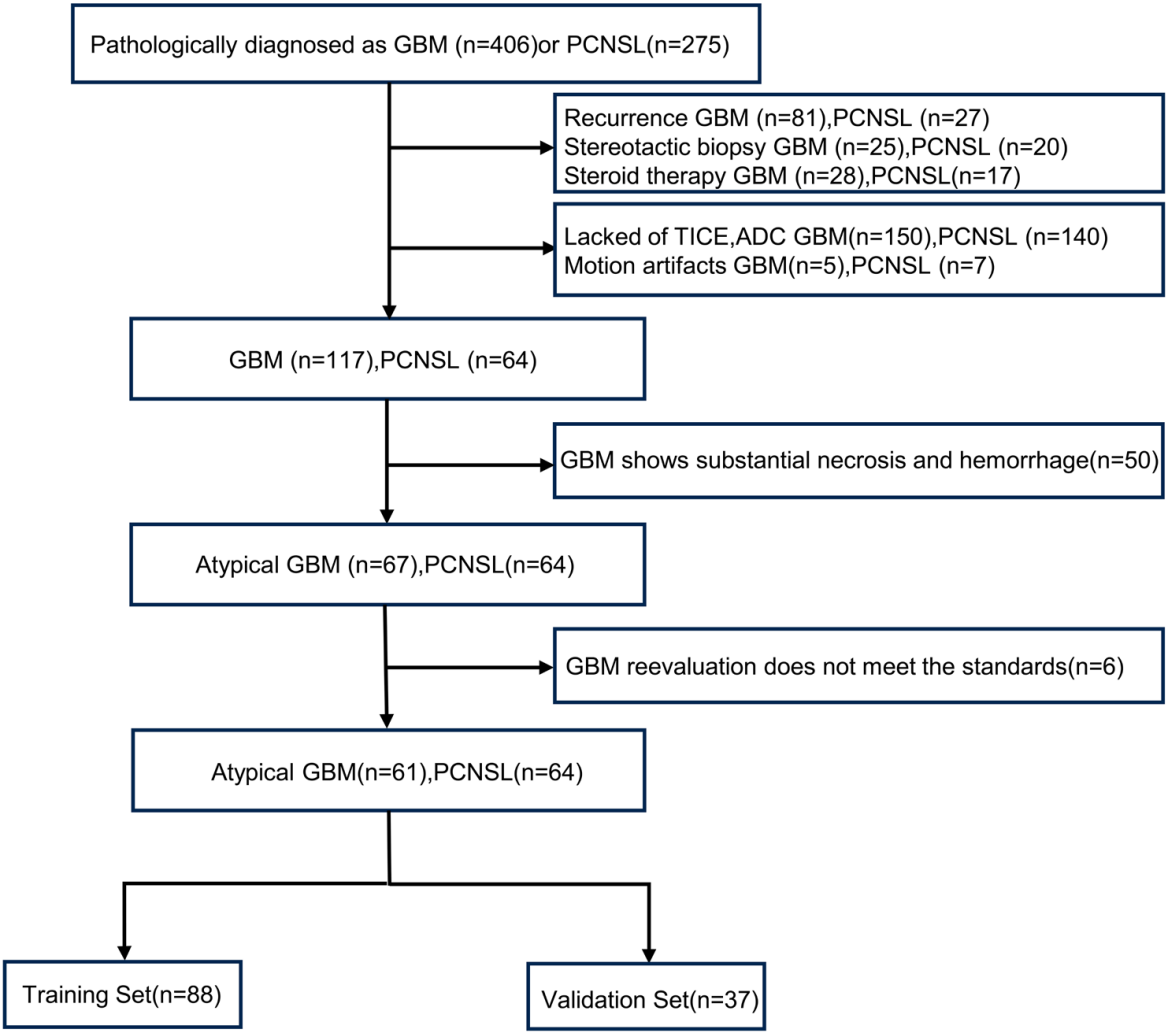
Flowchart of the patient enrolment process. ADC, apparent diffusion coefficient; GBM, glioblastoma; PCNSL, primary central nervous system lymphoma; T1CE, T1 contrast‐enhanced.

Atypical imaging features of GBM included an absence of necrosis or minimal necrosis. Pre‐treatment MRI of each GBM patient was independently evaluated by two radiologists (M.N.S. and D.D.F., 6 years and 16 years of imaging diagnosis experience) to identify atypical imaging features. Patients were classified into the atypical GBM group (67 patients) when both independent radiologists concurrently identified atypical imaging characteristics. Two additional neuroradiologists (D.Z. and C.Z.S., with 10 and 22 years of imaging diagnosis experience, respectively) provided their assessments for the 67 atypical GBM patients, and after a joint review, six patients with inconsistent judgments were further excluded. Ultimately, a total of 125 patients (61 atypical GBM, 64 PCNSL) were included in the study. Diagnosis of PCNSL was based on pathological examination. The patient enrollment process is depicted in Figure 1.

### 2.2 Image Preprocessing and Target Delineation

All patient images were exported from the PACS system in DICOM format. The images underwent N4 bias field correction and were resampled to a voxel size of 1 × 1 × 3 mm^3^. To enhance the contrast of the image, the image data is normalized to the 0–255 range. This normalization process is based on the gradient image, rather than the original image data. Specifically, it is achieved by calculating the mean and standard deviation of the image gradient. The T1WI, T2WI, and FLAIR images were registered to the T1‐CE image using ITK‐SNAP 3.8 software [11]. Following consultation with two neuroradiologists (M.N.S. and C.Z.S., 6 years and 22 years of imaging diagnosis experience), we excluded hemorrhagic lesions, which exhibit high signal intensity on both T1WI and T1‐CE images. Tumor regions of interest (ROI) were delineated on the T1‐CE sequences.

### 2.3 Segmentation of Subregions Within the Entire Tumor Based on K‐Means Clustering

**FIGURE 2:**
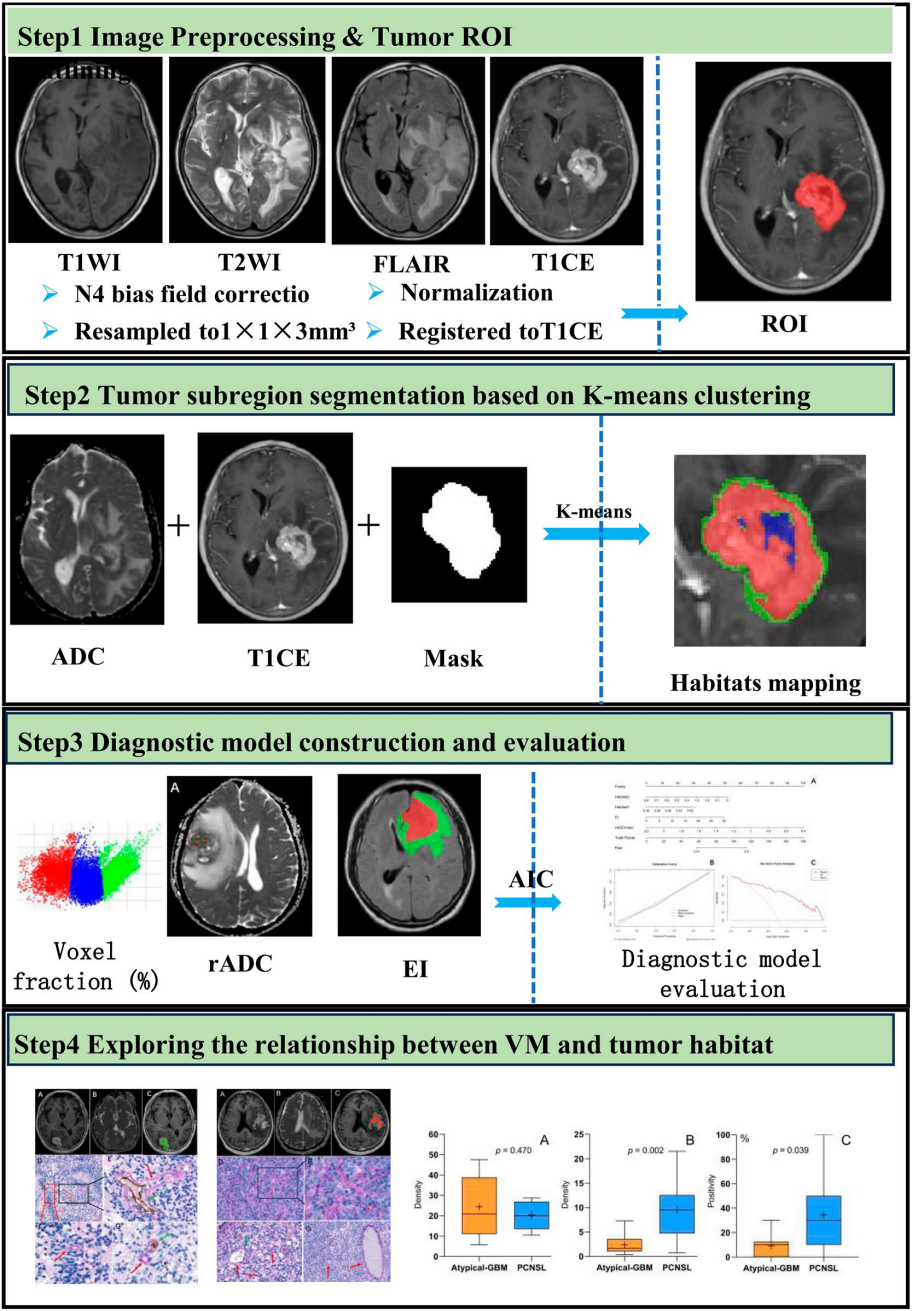
Workflow for research. High‐enhancement cellular habitat (Habitat 1, red) characterized by relatively high T1CE values and relatively low ADC values; low‐enhancement cellular habitat (Habitat 2, greener) characterized by relatively low T1CE values and relatively low ADC values; and nonviable tissue habitat (Habitat 3, blue) characterized by low T1CE values and relatively high ADC values. T1WI, T1‐weighted imaging; T2WI, T2‐weighted imaging; FLAIR: fluid attenuated inversion recovery.

Tumor habitat subregions based on the T1‐CE and ADC sequences were generated, employing the K‐means clustering method for unsupervised ROI segmentation. Subsequently, the segmented ecological subregions were mapped onto both the T1‐CE and ADC images. Similar to other studies [12, 13, 14, 15], we tested three, four, and five clusters to ensure that the results were interpretable, and ultimately chose three clusters. When calculating the number of clusters based on variables such as higher and lower for each pair of images, there are a total of four scenarios. For instance, T1‐CE and ADC can respectively represent high/low enhancement and high/low cellularity. In this study, regions of high enhancement and low cellularity were not identified. Three habitats were defined using two distinct T1‐CE and ADC feature maps: “high‐enhancement cellular habitat (Habitat 1)” characterized by relatively high T1‐CE values and relatively low ADC values; “low‐enhancement cellular habitat (Habitat 2)” characterized by relatively low T1‐CE values and relatively low ADC values; and “nonviable tissue habitat (Habitat 3)” characterized by low T1‐CE values and relatively high ADC values. Finally, the percentage of voxels for each habitat relative to the total number of tumor voxels (Habitat X voxels/Tumor voxels) was calculated (Figure 2).

### 2.4 CD34‐PAS Dual Staining Assay

CD34‐Periodic Acid‐Schiff (PAS)‐dual staining was performed as previously reported [16]. Standard immunohistochemical staining was performed on paraffin‐embedded tumor sections at a thickness of 5 μm for CD34. After this, the slides were rinsed with distilled water for 5 min and then incubated with PAS for 15 min. Following this incubation, the slides were counterstained with hematoxylin for 1 min and then covered with a slip. The slides were subsequently examined under a light microscope to detect CD34 and PAS signals. Two independent observers (M.N.S. and X.Y.H., 6 and 14 years of experience), who were unaware of the outcomes, examined the whole slide for the presence of VM by identifying CD34‐negative and PAS‐positive vessels, as evaluated according to the criteria set by Folberg [7], endothelial vessels were CD34+/PAS+, subsequently reviewed by a neuro‐oncology pathologist with 20 years of experience (H.N.L.). Slides were digitized at 400 × absolute magnification using the KFBIO Pathology System, and the digital images were analyzed using K‐Viewer (version 1.7, KFBIO, Yuyao, CHN).

### 2.5 VM Quantification

In K‐Viewer, we first excluded necrotic tumor tissue from the analysis area, then randomly placed 10 boxes measuring 550 × 500 μm within this area, making sure that the spacing between the boxes was at least 550 μm [17]. For each box, the number of discrete VM and endothelial vessels was counted. Discrete VM include straight channels, arrangements of parallel straight channels, arcs (incompletely closed loops), arcs with branching, and closed loops. Densities for discrete VM and endothelial vessels were calculated as the mean count/mm^2^. Additionally, we assessed the presence or absence of continuous VM, such as straight channels that cross‐link, closed loops, and networks, within each of the 10 areas. The vascular counts were conducted by three independent observers (M.N.S., H.W. and X.Y.H., 6, 15, and 14 years of experience) and were subsequently reviewed by a neuro‐oncology pathologist with 20 years of experience (H.N.L.). The average of the vascular counts from the three observers was taken as the final result.

### 2.6 Statistical Analysis

Statistical analyses were performed using IBM SPSS Statistics for Windows, version 27.0 (IBM Corporation, Armonk, NY, USA), GraphPad Prism (version 9.1.1, GraphPad Software, Boston, MA, USA), and R (version 4.2.1, R Foundation for Statistical Computing). Datasets were tested for normality using the Shapiro–Wilk test. Normally distributed data are presented as mean ± standard deviation. Non‐normally distributed data are expressed as the median (interquartile range). Mann–Whitney *U* test or *t*‐test was used to compare continuous variables between the atypical GBM and PCNSL group. Chi‐square test was used to compare sex, tumor location, and initial symptoms between the atypical GBM and PCNSL group. Two‐tailed Wilcoxon matched‐pairs signed rank tests were utilized to assess the median differences in the densities of VM and the positivity of continuous VM between the atypical GBM and PCNSL group. The random selection of patients for CD34‐PAS dual staining was conducted using SPSS software. *p* < 0.05 was considered statistically significant. Parameters showing significant differences between the two tumor groups were evaluated using receiver operating characteristic (ROC) curve analysis to calculate the area under the curve (AUC), which indicates the ability of these parameters to differentiate between the two tumor types. The maximum Youden index was obtained from the ROC curves to determine the optimal cutoff values for calculating the sensitivity and specificity.

The univariate logistic regression analysis was performed on the parameters with significant differences between the two tumor groups. Variables with a *p* < 0.05 from the univariable logistic regression analysis were included in the multivariable logistic regression model. To examine multicollinearity among the predictor variables and prevent overfitting of the model, the Akaike Information Criterion (AIC) method was employed to select the optimal model. Using the indicators derived from this model, a nomogram was constructed. The discriminatory ability of the model was then assessed using the ROC curve. Next, the calibration of the model was evaluated through the calibration curve, with bias corrected by performing 1000 bootstrap resamples. Calibration was assessed with the Hosmer‐Lemeshow goodness‐of‐fit test, where a *p* greater than 0.05 indicates consistent calibration with the model. Finally, the clinical effectiveness of the model was evaluated using decision curve analysis (DCA). Multiple linear regression analysis was conducted to investigate the relationships between the proportions of Habitat 1, Habitat 2, and Habitat 3 voxels with the following variables: the densities of endothelial vessels and discrete VM, and the positive rate of continuous VM.

## 3 Results

### 3.1 Patient Characteristics

One hundred twenty‐five patients (61 atypical GBM and 64 PCNSL), divided into a training set (88) and a validation set on a 7:3 basis. There was a significant difference in the mean age between the atypical GBM group (mean ± standard deviation: 50.6 ± 15.0 years) and the PCNSL group (mean ± standard deviation: 56.9 ± 12.7 years). In the atypical GBM group, there were 33 men and 28 women, while in the PCNSL group, there were 30 men and 34 women, with no significant difference in the sex distribution between the two groups (*p* = 0.419). In the atypical GBM group, 30 patients involved midline structures, compared to 38 patients in the PCNSL group, with no significant difference (*p* = 0.253). There was a statistically significant difference in the tumor diameter between the atypical GBM and PCNSL groups. The median tumor diameter in the atypical GBM group was 47.3 mm, with the first quartile and third quartile being 33.7 and 58.3 mm, respectively. In the PCNSL group, the median tumor diameter was 31.1 mm, with the first quartile and third quartile being 22.2 and 49.9 mm, respectively. A summary of the patient and imaging characteristics in the atypical GBM and PCNSL groups is provided in Table 1 and Figure S3.

**TABLE 1:** Information about patients with atypical GBM and PCNSL.

Parameter	Atypical GBM (*n* = 61)	PCNSL (*n* = 64)	*p*
Age (years)			
Mean ± SD	50.6 ± 15.0	56.9 ± 12.7	0.013*
Range	18–84	20–88	
Gender (%)			0.419
Male	33 (54.1%)	30 (46.9%)	
Female	28 (45.9%)	34 (53.1%)	
Headaches (%)	30 (49.2%)	26 (40.6%)	0.336
Located midline (%)			0.253
Yes	30 (49.2%)	38 (59.4%)	
No	31 (50.8%)	26 (40.6%)	
Tumor diameter (mm)			0.002*
*M* (*P*_25_, *P*_75_)	47.3 (33.7, 58.3)	31.1 (22.2, 49.9)	
Habitat 1			< 0.001*
Mean ± SD	39.5% ± 14.2%	54.3% ± 17.7%	
Habitat 2			< 0.001*
Mean ± SD	52.5% ± 13.3%	41.5% ± 16.3%	
Habitat 3			0.025
*M* (*P*_25_, *P*_75_)	10.7% (6.9%, 13.0%)	5.9% (2.3%, 11.4%)	
EI			< 0.001
*M* (*P*_25_, *P*_75_)	2.008 (1.345, 3.238)	3.445 (2.182, 5.233)	
rADC_mean_			< 0.001
*M* (*P*_25_, *P*_75_)	1.201 (1.025, 1.399)	0.968 (0.856, 1.112)	
rADC_max_			< 0.001
*M* (*P*_25_, *P*_75_)	1.580 (1.356, 1.899)	1.271 (1.086, 1.458)	
rADC_min_			< 0.001
*M* (*P*_25_, *P*_75_)	0.993 (0.804, 1.112)	0.803 (0.660, 0.891)	
rADC_dif_			< 0.001
*M* (*P*_25_, *P*_75_)	0.624 (0.496, 0.856)	0.461 (0.345, 0.598)	

*Note*: *indicates that the *p* value of this parameter is less than 0.05.

Abbreviations: EI, edema index; GBM, glioblastoma; PCNSL, primary central nervous system lymphoma; rADCdif, relative (max–min) apparent diffusion coefficient; rADCmax, relative maximum apparent diffusion coefficient; rADCmean, relative mean apparent diffusion coefficient; rADCmin, relative minimum apparent diffusion coefficient.

### 3.2 Tumor Habitat Analysis

The interobserver ICC for ROI was 0.990. The mean voxel fraction of high‐enhancement cellular habitat (Habitat 1) in the PCNSL group was 54.3%, which was significantly higher than the atypical GBM group (39.5%); in contrast, the mean voxel fraction of low‐enhancement cellular habitat (Habitat 2) was 52.5% in the atypical GBM group, significantly higher than the PCNSL group (41.5%); the median voxel fractions of nonviable tissue habitat (Habitat 3) were 10.7% in the atypical GBM group and 5.9% in the PCNSL group, with a statistically significant difference between the two groups (Table 1 and Figure S3).

### 3.3 EI, and rADC Analysis

The median EI of patients in the PCNSL group was 3.445, significantly higher than the atypical GBM group (2.008). The distribution of tumor habitats and EI in the atypical GBM and PCNSL groups is shown in Table 1 and Figure S3. The rADCmin, rADCmax, rADCmean, and rADCdif values in the atypical GBM group (0.993, 1.580, 1.201, and 0.624) are significantly higher than those in the PCNSL group (0.803, 1.271, 0.968, and 0.461). As shown in Table 1 and Figure S4, the rADCmin, rADCmax, rADCmean, and rADCdif measurements for patients with atypical GBM and PCNSL are presented and compared. The representative tumor habitat for atypical GBM and PCNSL is shown in Figure S5.

### 3.4 Diagnostic Model Evaluation

**FIGURE 3:**
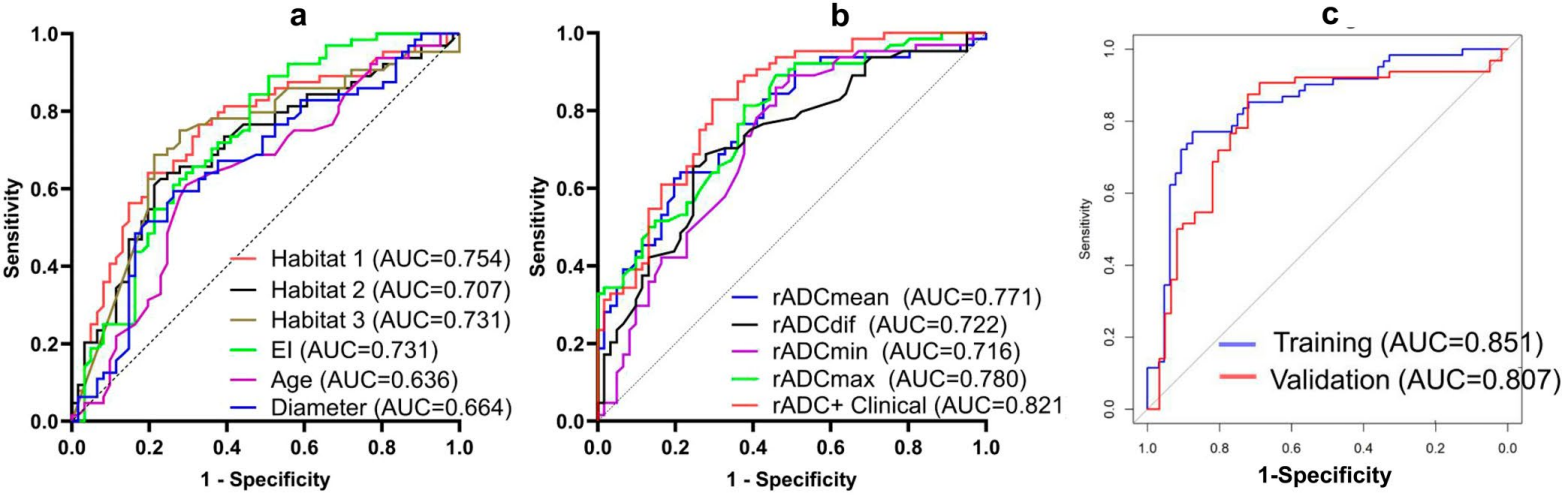
(a) Receiver operating characteristic (ROC) curves for tumor habitat, age, diameter, and EI for the differentiation between atypical GBM and PCNSL. (b) ROC curves for rADCmean, rADCmax, rADCmin, and rADCdif for the differentiation between atypical GBM and PCNSL. (c) Predict the ROC curves of the model in the training and validation sets.

**FIGURE 4:**
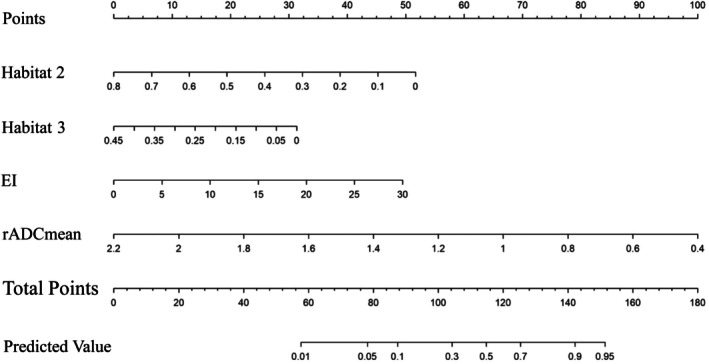
The nomogram for distinguishing atypical GBM from PCNSL.

**FIGURE 5:**
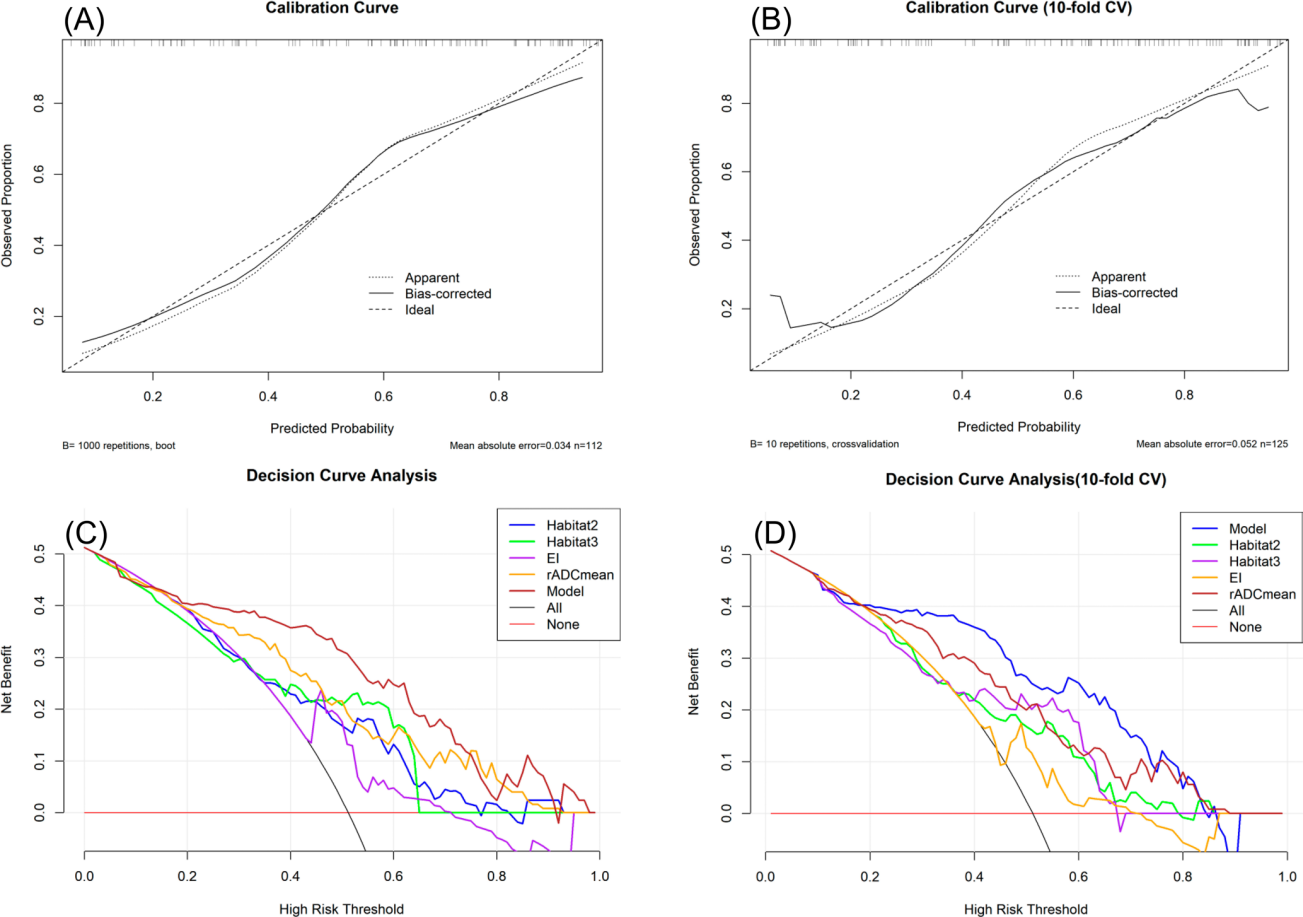
Calibration curves of the model in the training set (A) and validation set (B). Decision curve analysis of predictive and one‐factor models in the training set (C) and validation set (D).

The univariate logistic analysis revealed that age, tumor diameter, Habitat 1, Habitat 2, Habitat 3, EI, rADCmean, rADCmax, rADCmin, and rADCdif all exhibited statistical significance (Table 2). Among these, rADCmax demonstrated the highest diagnostic performance with an AUC of 0.780 (95% CI: 0.701–0.859) (Table 3 and Figure 3). The optimal model, selected using the AIC method, includes four independent factors: Habitat 2, Habitat 3, EI, and rADCmean (Figure 4), with tolerance values ranging from 0.914 to 0.978 and VIF values ranging from 1.022 to 1.095. This model demonstrated good diagnostic performance in both the training and validation sets, with an AUC of 0.851 (95% CI: 0.781–0.921) for the training set and 0.807 (95% CI: 0.724–0.889) for the validation set (Figure 3). The calibration curve analysis showed strong consistency between the actual and ideal curves in both the training sets (Hosmer–Lemeshow χ^2^ = 12.812, *p* = 0.119) and validation sets (Hosmer‐Lemeshow χ^2^ = 9.009, *p* = 0.173) (Figure 5). Finally, the DCA demonstrated that the combined model offers a net benefit superior to that of the univariate model, within a threshold probability range of 0.15–0.78, thereby providing greater clinical benefit for patients (Figure 5).

**TABLE 2:** Univariable logistic regression analysis for atypical GBM and PCNSL group.

Parameter	*B*	SE	OR	95% CI	*p*
Age	0.033	0.014	1.033	1.006–1.061	0.016
Diameter	−0.036	0.011	0.964	0.943–0.986	0.001
Habitat 1	5.898	1.361	364.5	25.3–5246.4	< 0.001
Habitat 2	−5.110	1.396	0.006	0.000–0.093	< 0.001
Habitat 3	−9.500	3.225	0.000	0.000–0.042	0.003
EI	0.110	0.060	1.116	0.993–1.255	0.026
rADCmean	−4.512	1.008	0.011	0.002–0.079	< 0.001
rADCmax	−3.634	0.771	0.026	0.006–0.120	< 0.001
rADCmin	−3.546	0.958	0.029	0.004–0.188	< 0.001
rADCdif	−3.711	0.961	0.024	0.004–0.161	< 0.001

Abbreviations: EI, Edema index; GBM, glioblastoma; PCNSL, primary central nervous system lymphoma; rADCdif, relative (max–min) apparent diffusion coefficient; rADCmax, relative maximum apparent diffusion coefficient; rADCmean, relative mean apparent diffusion coefficient; rADCmin, relative minimum apparent diffusion coefficient.

**TABLE 3:** Accuracy of Habitat, age, tumor diameter, EI and rADC in diagnosing atypical GBM and PCNSL.

Parameter	AUC (95% CI)	Sensitivity	Specificity	Cut‐off value
Habitat 1	0.754 (0.667–0.834)	0.641	0.803	48.85%
Habitat 2	0.709 (0.615–0.799)	0.609	0.787	43.40%
Habitat 3	0.731 (0.639–0.822)	0.787	0.687	2.95%
EI	0.731 (0.642–0.820)	0.844	0.541	0.936
Age	0.636 (0.538–0.735)	0.609	0.705	56.50
Diameter	0.664 (0.567–0.760)	0.594	0.738	35.50
rADC_mean_	0.776 (0.695–0.856)	0.787	0.641	1.015
rADC_max_	0.780 (0.701–0.859)	0.557	0.891	1.552
rADC_min_	0.717 (0.626–0.808)	0.541	0.859	0.946
rADC_dif_	0.723 (0.634–0.812)	0.721	0.703	0.546

Abbreviations: EI, edema index; rADCmax, relative maximum apparent diffusion coefficient; GBM, glioblastoma; PCNSL, primary central nervous system lymphoma; rADCdif, relative (max–min) apparent diffusion coefficient; rADCmean, relative mean apparent diffusion coefficient; rADCmin, relative minimum apparent diffusion coefficient.

### 3.5 Vasculogenic Mimicry in Atypical GBM and PCNSL Group

**FIGURE 6:**
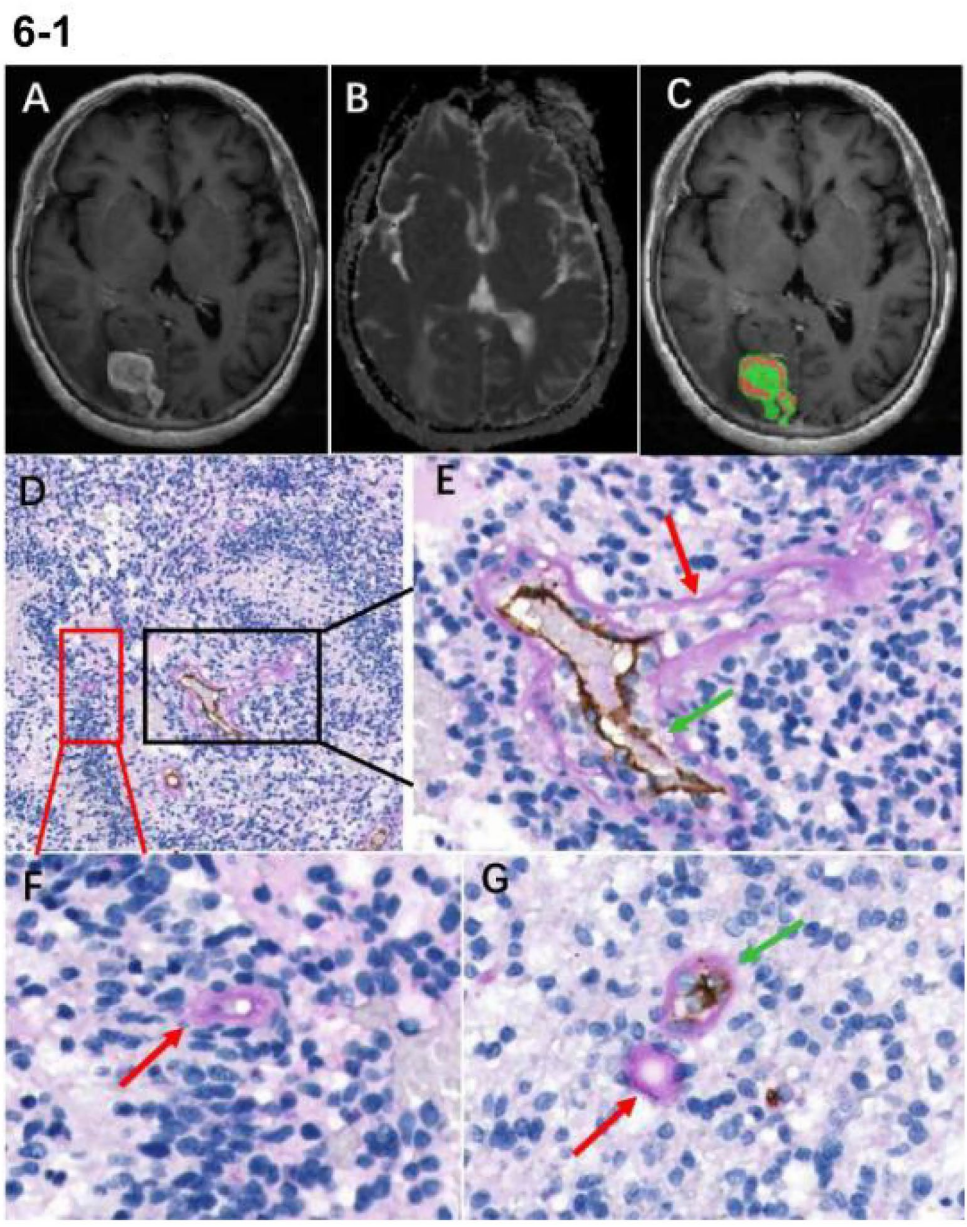
(A–C) The images of T1CE, ADC, and tumor habitat from a 71‐year‐old female patient diagnosed with GBM. Habitat 1 voxel percentage of 40.8%, habitat 2 voxel percentage of 59.2%. A (D–G) Tissue stains: CD34 and PAS staining. Endothelial cells were identified using anti‐CD34 immunohistochemical staining (brown product). PAS positivity (purple‐red) with an absence of CD34‐positive cell reactions indicates the presence of VM structures, the presence of CD34+/PAS+ staining signifies endothelial vessels. (D) (×100) Only a limited number of CD34−/PAS+ VM structures are observed (highlighted in red and black boxes). The boxed area in D is magnified in E and F. (E–G) (×400) PAS‐positive patterns with a hollow channel (indicated by a red arrow) and CD34+/PAS+ endothelial vessels (indicated by a green arrow) can be seen together, the VM structures are continuous with the endothelial vessels (E).

**FIGURE 7:**
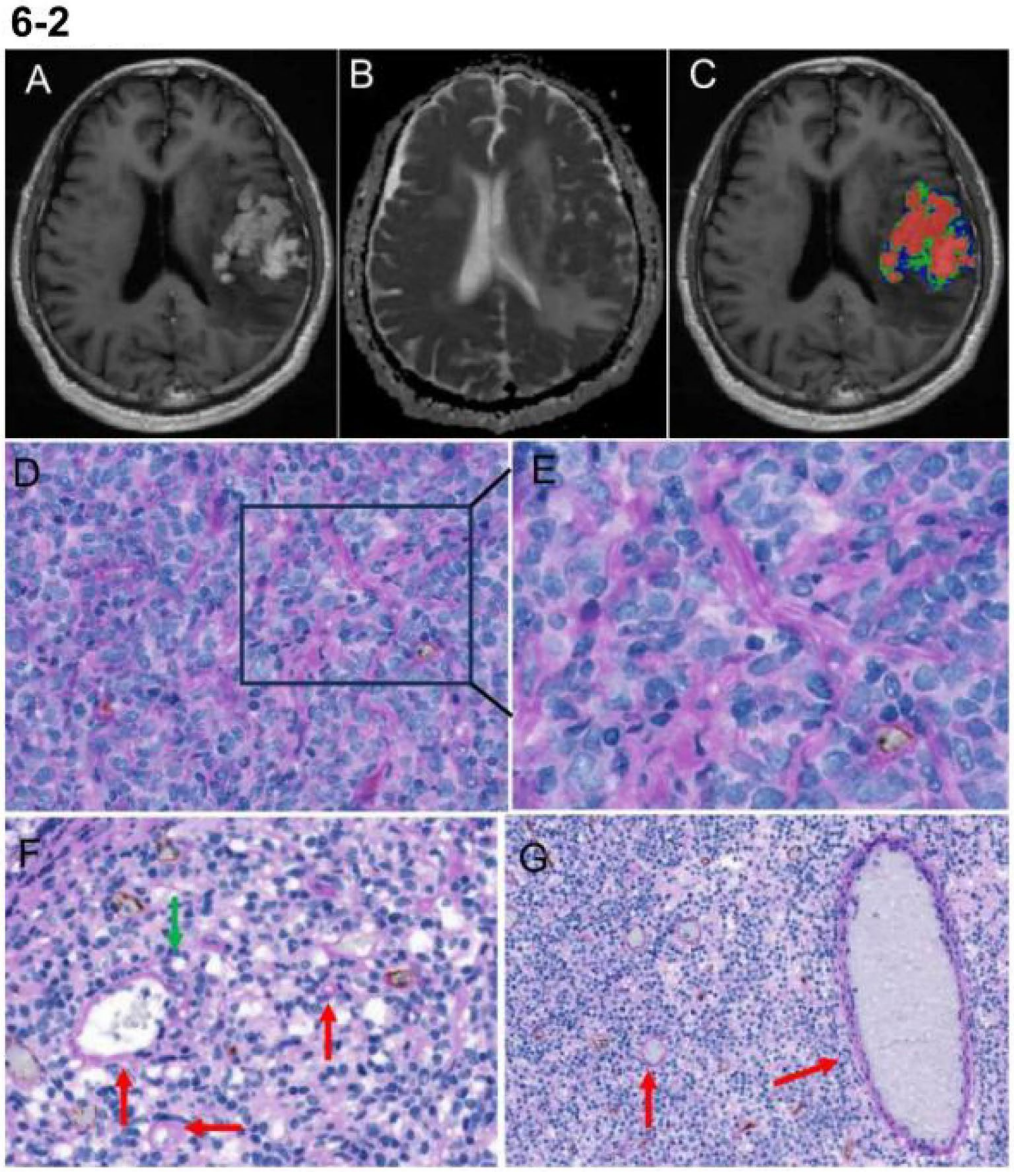
(A–C) The images of T1CE, ADC, and tumor habitat from a 65‐year‐old female patient diagnosed with PCNSL. Habitat 1 voxel percentage of 58.1%, habitat 2 voxel percentage of 21.9% and habitat 3 voxel percentage of 20.0%. (D) (×100) Abundant network‐like VM structures are observed. The boxed area in D is magnified in E. (E) (×400) There are red‐stained network‐like CD34−/PAS+ structures with a hollow channel. (F) (×400) and (G) (×200) Contain a larger number of PAS‐positive tubular structures (indicated by the red arrow). The circular structures formed by tumor cells are covered by a PAS red‐stained basement membrane (indicated by the green arrow).

**FIGURE 8:**
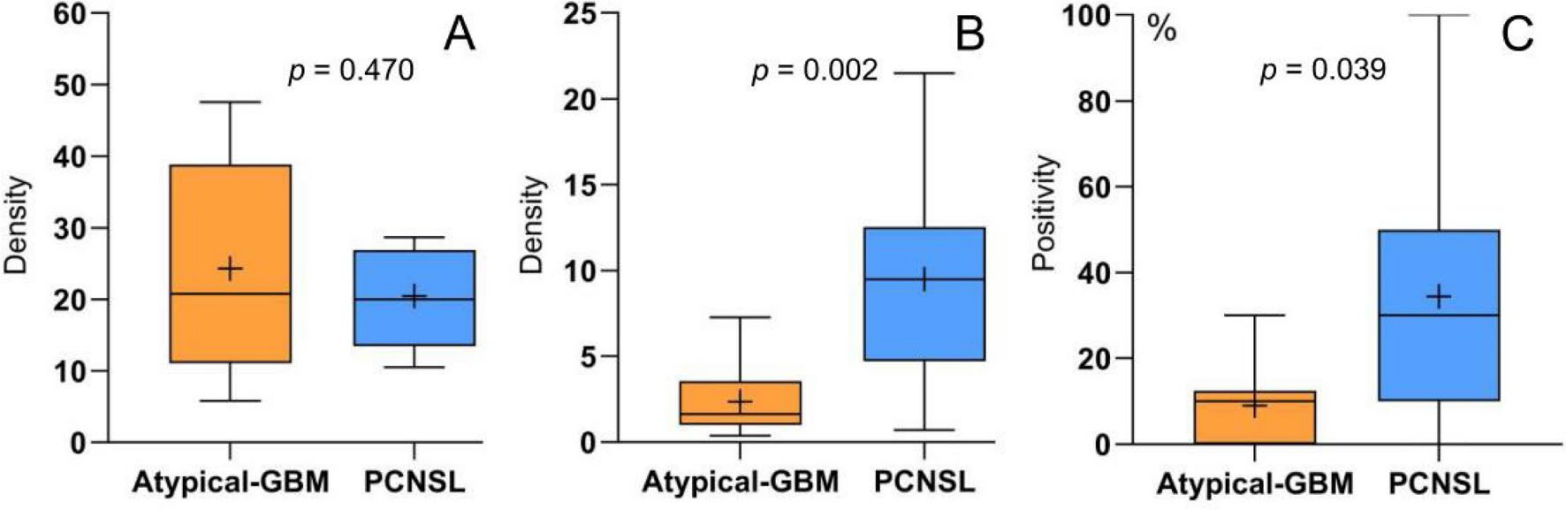
A Comparison of endothelial vessel, discrete VM, and continuous VM between atypical GBM and PCNSL. “+” represents the average value. (A) The average endothelial vessel density in the atypical GBM groups was slightly higher than that in the PCNSL group. (B) The discrete VM in the PCNSL group was significantly higher than that in the atypical GBM group. (C) The continuous VM in the PCNSL group also exhibits a significantly higher value compared to the atypical GBM group.

The study randomly selected 19 patients (10 with atypical GBM and 9 with PCNSL) for CD34‐PAS dual staining. The results from the CD34‐PAS dual staining revealed that most intratumoral vessels exhibited a positive reaction for CD34 on the luminal surface and a positive reaction for PAS on the vessel wall. Notably, we observed the presence of PAS+/CD34‐ tubular structures containing red blood cells, which are recognized as typical structures of VM based on previously reported criteria [18]. These PAS‐positive and CD34‐negative patterns were found in the viable areas of the tumor, where they formed different morphological patterns (Figures 6 and 7). The median density of discrete VM in the PCNSL group (9.1 vessels/mm^2^) was significantly higher than that in the GBM group (1.6 vessels/mm^2^). Similarly, the positivity rate for continuous VM was also greater in the PCNSL group (30%) compared to the GBM group (10%). The mean density of endothelial vessels in the atypical GBM group (24.3 vessels/mm^2^) was higher than in the PCNSL group (20.4 vessels/mm^2^); however, this difference was not statistically significant (*p* = 0.470) (Table S4 and Figure 8). The distribution of tumor habitats in the 19 patients is shown in Figure S6.

### 3.6 Correlation of Tumor Habitat With Pathologic Features

The multiple linear regression analysis indicated that the densities of discrete VM and endothelial vessels, as well as the positivity rate of continuous VM, were positively correlated with the voxel proportion of Habitat 1 (Adjusted *R* ^2^ = 0.644, *F* = 11.872, D‐W = 1.899). In contrast, these variables were negatively correlated with the voxel proportion of Habitat 2 (Adjusted *R* ^2^ = 0.477, *F* = 6.462, D‐W = 1.874). The impact of discrete VM density on the voxel proportion of Habitat 1 was statistically significant (*β* = 0.692, *t* = 4.336), as was the impact of endothelial vessel density (*β* = 0.414, *t* = 2.913), (Table S1). Among the three variables, only the density of discrete VM had a statistically significant impact on the voxel proportion of Habitat 2 (*β* = −0.579, *t* = −2.990), (Table S2). The positivity rate of continuous VM shows a significant correlation with the voxel proportion of Habitat 3 (*β* = −0.708, t = 3.519), (Table S3).

## 4 Discussion

The tumor habitats provide a more accurate reflection of the tumor heterogeneity. We developed a model which includes four independent parameters: Habitat 2, Habitat 3, EI, and rADCmean. This model effectively differentiates atypical GBM from PCNSL. In this study, the median voxel fractions of nonviable tissue in atypical GBM were lower than that in the study by Choi et al. [6], who defined atypical GBM as less than 13% necrosis of the enhancing tumor. The findings of Bathla et al. [19] suggest that excellent discrimination between PCNSL and GBM can be achieved through machine learning. Similarly, the study by Suh et al. [20] found that large‐scale radiomics combined with a machine‐learning algorithm can effectively differentiate PCNSL from atypical GBM and offers better diagnostic performance than human radiologists and ADC values. Lee et al. [21] discovered that analyzing the combined permeability and perfusion metrics from a single dynamic susceptibility contrast MRI (DSC‐MRI) acquisition enhances the diagnostic accuracy in distinguishing PCNSL from GBM, outperforming single‐parameter nCBV analysis. Compared with DSC‐MRI, T1‐CE and ADC are routinely used in the majority of patients with brain tumors; therefore, tumor habitats based on T1‐CE and ADC derivation have better clinical utility.

The mechanism underlying MRI contrast enhancement in intracranial tumors involves the incomplete blood–brain barrier and increased vascular permeability. This allows the contrast agent to leak from the blood vessels and accumulate in the interstitial spaces, thereby shortening the T1 relaxation time of the tumor tissues [22, 23]. We found that the endothelial vessel density in the atypical GBM group was higher than that in the PCNSL group. This finding aligns with the results of another study [24]; the study found that the microvessel density labeled by anti‐CD34 in the PCNSL group was much lower than that in the GBM group. The correlation analysis revealed that the voxel ratio of Habitat 1 exhibited a positive correlation with endothelial vessel density. In contrast, the voxel ratio of Habitat 2 displayed a negative correlation with endothelial vessel density, theoretically resulting in a higher voxel ratio of the enhanced tumor area in the atypical GBM group compared to the PCNSL group. However, our study found that the high‐enhancement cellular habitat voxel ratio is higher in the PCNSL group compared to that in the atypical GBM group, whereas the low‐enhancement cellular habitat voxel ratio is higher in the atypical GBM group compared to that in the PCNSL group. This may be related to differences in the growth patterns of the two types of tumors. PCNSL cells exhibit angiocentricity and are characterized by a perivascular arrangement known pathologically as a “cuffing” infiltration. This angiocentric nature of PCNSL results in extensive infiltration around blood vessel walls, leading to widespread disruption of the BBB and increased vascular permeability. In contrast, no such organized structural arrangement has been observed in GBM cells [25]. This ultimately contributes to significant differences in voxel ratios of high‐enhancement and low‐enhancement cellular habitats between the two types of tumors.

VM [26] is a vessel‐like structure found in vascular‐dependent solid tumors, formed through self‐deformation of highly invasive tumor cells and extracellular matrix remodeling. It serves as a distinct blood supply source contributing to the progression of highly invasive tumors and is associated with tumor invasion, metastasis, and prognosis [26, 27]. Maniotis et al. [28] first reported the VM structure when studying the microcirculation patterns of human choroidal melanoma. There are two types of VM [29]: tubular and matrix. Tubular VM is composed of tumor cells rather than endothelial cells, while matrix VM is primarily composed of extracellular matrix rather than tumor cells. Yue et al. [30] confirmed the presence of VM in human GBM in 2005. Maddison et al. [17] found that the VM content is significantly lower compared to neoangiogenesis, both in primary and recurrent GBM. In a study by Huang [31] that used immunohistochemistry and CD34/PAS dual staining, VM structures were detected in only 34 out of 127 patients with GBM (26.8%). Currently, there are limited data on the relationship between PCNSL and VM. Qi et al. [32] conducted CD34/PAS dual staining on 96 patients with PCNSL and found the presence of VM structures in all tumors. Additionally, Qi et al. compared the correlation between VM content and enhancement index, revealing significantly higher enhancement index levels in strongly VM positive groups compared to weakly VM positive groups.

In the current study, the proportion of high‐enhancement cellular habitat (Habitat 1) voxels in the PCNSL group was higher than that in the atypical GBM group, whereas the atypical GBM group showed a higher proportion of low‐enhancement cellular habitat (Habitat 2) voxels compared to that in the PCNSL group. The histopathological evaluation indicated that the quantities of both discrete and continuous VM structures are significantly higher in the PCNSL group compared to the atypical GBM group. The increased number of VM structures resulted in greater contrast agent extravasation in the PCNSL group, which was reflected in the tumor habitat as a higher proportion of Habitat 1 voxels in the PCNSL group compared to the atypical GBM group. Multiple regression analysis indicates that the voxel fraction of Habitat 1 was positively correlated with the density of discrete VM. Furthermore, among the three variables analyzed, only the density of discrete VM significantly impacted the voxel proportion of Habitat 2. This suggests that VM structures may represent one of the important pathological mechanisms contributing to the differences in tumor habitats between the atypical GBM and PCNSL groups.

In this study, the proportion of nonviable tissue habitat voxel fractions (Habitat 3) was significantly higher in the atypical GBM groups compared to that in the PCNSL group. We believe this may be related to the angiophilic nature of PCNSL, where increased vascular permeability leads to enhanced extravasation of nutrients, facilitating the growth of tumor cells around the vessels to obtain nourishment. Although PCNSL exhibits higher vascular permeability [33], the microvascular density is lower than that of GBM [24], which may restrict the amount of nutrients leaking from damaged vessel walls. The presence of VM structures could help explain why the voxel fractions of Habitat 3 are lower in the PCNSL group. VM connected to vessels increases the tumor vascular supply, allowing distant tumor cells to receive nutrients. In contrast, GBM has lower vascular permeability than PCNSL, resulting in reduced nutrient extravasation. Additionally, GBM exhibits a lower VM content [17], further decreasing oxygen and nutrient supply to tumor cells distant from the vessels [34, 35], which may increase the proportion of necrotic voxel regions compared to those in PCNSL.

In the study by Hung et al. [36], no significant difference was found in the ADC values of PTBE between the GBM and PCNSL groups. In contrast, the study by Sha et al. [37] demonstrated that texture analysis of peritumoral edema could effectively differentiate between GBM and PCNSL. Unlike previous two‐dimensional studies [38, 39], the research offers a more comprehensive understanding of the relationship between the tumor and PTBE. Furthermore, this approach eliminates the subjective biases associated with ROI selection, leading to more concise and reliable conclusions. In our study, the EI of the PCNSL group was significantly higher than that of the atypical GBM group. This difference was linked to the greater disruption of the BBB in PCNSL. In brain tumors, peritumoral edema is associated with BBB disruption, where the BBB is either partially or completely compromised in the tumor region, allowing water and plasma proteins to leak from capillaries into the surrounding tissue [37]. As a result, the more extensive BBB disruption observed in PCNSL leads to a higher EI compared to atypical GBM.

## 5 Limitations

Firstly, our study had a small sample size and limited observational metrics, which may have introduced bias in the results. It is necessary to expand the sample size and refine the study design. Secondly, the diagnostic performance of tumor habitat was not compared with other commonly used advanced MRI parameters such as rCBV. Therefore, larger studies are needed in the future.

## 6 Conclusions

The tumor habitat constructed using T1‐CE and ADC sequences may be capable of distinguishing atypical GBM from PCNSL. We developed a model that incorporates the parameters Habitat 2, Habitat 3, EI, and rADCmean. This model exhibits strong distinguishing power and can accurately differentiate atypical GBM from PCNSL. Additionally, the differences in VM content are one of the pathological mechanisms underlying the variations in tumor habitats between atypical GBM and PCNSL.

## Supporting information


**Data S1:** Supporting Information.
