# Supplementary material for: Tumor Habitats Based on Multiparametric MRI Distinguish Atypical Glioblastoma From Primary Central Nervous System Lymphoma: Imaging‐Pathologic Correlation
Source: J Magn Reson Imaging. 2025 Aug 20;63(1):142–54. doi: 10.1002/jmri.70080 (PMC12706713; doi:10.1002/jmri.70080)
Supplement: Supplementary file 1 — Data S1: Supporting Information. [file JMRI-63-142-s001.pdf]

Supplementary Materials for

Tumor habitats based on multiparametric MRI distinguish atypical glioblastoma from primary central nervous system lymphoma: imaging-pathologic correlation

MRI parameters

MR images were acquired using a 3.0-T (Signa HDxt; GE Healthcare) or 1.5-T (Achieva; Philips Medical Systems) MR scanner with an 8-channel head coil. The imaging sequences included T1-weighted imaging (T1WI), T2-weighted imaging (T2WI), T2-fluid-attenuated inversion recovery (T2-FLAIR), T1 contrast-enhanced (T1CE), and diffusion-weighted imaging (DWI). The parameters for T1WI were TR (repetition time) of 488-1900 ms and TE (echo time) of 15-24 ms. The parameters for T2WI were TR of 4480-6000 ms and TE of 120 ms. The parameters for T2-FLAIR were TR of 7780-9480 ms, TE of 120-135 ms, FOV (field of view) of 240×240 mm2, matrix of 256×256, slice thickness of 5.5 mm (with a gap of 1 mm), and NEX (number of excitations) of 1. The parameters for DWI were TR of 2262-6000 ms, TE of 74.7-75 ms, matrix of 256×256, slice thickness of 5.5 mm, FOV of 240×240 mm, and b-values of 0 and 800/1000 s/mm2.

Tumor diameter measurement and localization determination

The measurement of tumor maximum diameter was conducted by three experienced radiologists (MN.S, XJ.Y and XY.H), who independently measured the maximum cross-sectional tumor diameter using T1CE images. The average of these three measurements was used as the tumor's maximum diameter. Similarly, determination of tumor midline position was assessed by the aforementioned three doctors. In cases of disagreement, a radiologist with 22 years of experience (CZ.S) conducted a review.

Relative ADC measurement

Three or more circular Regions of Interest (ROIs), with areas ranging from 5 to 20 mm^2, were placed on the solid component of the tumor. The ADC values obtained from these ROIs were used to calculate the minimum (ADCmin) and maximum (ADCmax) values. The difference between these two values, referred to as ADCdif, was calculated by subtracting ADCmin from ADCmax. In addition, a large ROI was drawn to cover the largest axial cross-section of the tumor, while excluding areas of calcification, bleeding, and necrosis. This large ROI was then used to compute the mean ADC value, denoted as ADCmean. As reported in the study by Hagiwara et al[1], to reduce variability induced by different scanners, relative ADC (rADC) was calculated by normalizing the ADC maps using the mean value of normal-appearing white matter in the contralateral hemisphere. This approach helps minimize the influence of scanner differences. For the calculation, three spherical volumes of interest (VOIs), each 5 mm in diameter, were placed in the central semiovale at the anterior, middle, and posterior regions, approximately 3 mm above the upper end of the lateral ventricle. These three VOIs were then combined into a single ROI to extract the average ADC value of the normal-appearing white matter. We normalized the values of ADCmin, ADCmax, ADCmean, and ADCdif to the contralateral normal-appearing white matter. This process yielded the corresponding relative values: rADCmin, rADCmax, rADCmean, and rADCdif. The ADC measurement was performed using the off-line software RadiAnt DICOM Viewer as shown in Supplementary_Fig.1.

Edema Index Measurement

Tumor voxels was assessed using the T1CE sequence, whereas the PTBE voxels, which encompasses the tumor, was determined using T2-FLAIR sequences. PTBE primarily affects the

white matter around the tumor, exhibiting a low signal on T1WI and a high signal on T2-FLAIR images. Consequently, in this study, the PTBE voxels was calculated by subtracting the T1CE volume from the T2-FLAIR voxels (Supplementary_Fig.2). In this study, the peritumoral EI was utilized evaluate the degree of PTBE, EI = (Voxels tumor + Voxels edema) /Voxels tumor[2]. The EI measurement was performed using the off-line software ITK-SNAP 3.8.

[1] HAGIWARA A, OUGHOURLIAN T C, CHO N S, et al. Diffusion MRI is an early biomarker of overall survival benefit in IDH wild-type recurrent glioblastoma treated with immune checkpoint inhibitors [J]. Neuro-oncology, 2022, 24(6): 1020-8.

[2] QU S, HU T, QIU O, et al. Effect of Piezo1 Overexpression on Peritumoral Brain Edema in Glioblastomas [J]. AJNR American journal of neuroradiology, 2020, 41(8): 1423-9.

Supplementary_Table 1. Multiple linear regression analysis between VM, endothelial vessels and the proportions of Habitat 1 voxels.

Habitat 1 voxels	Standardized Coefficients Beta	*t*	*P*
Discrete VM	0.692	4.336	<0.001*
Continuous VM	0.110	0.682	0.506
Endothelial vessels	0.414	2.913	0.011*
F	11.872		
Adjusted R^2	0.644		
D-W	1.899		

Supplementary_Table 2. Multiple linear regression analysis between VM, endothelial vessels and the proportions of Habitat 2 voxels.

Habitat 2 voxels	Standardized Coefficients Beta	*t*	*P*
Discrete VM	-0.579	-2.990	0.009*
Continuous VM	-0.239	-1.223	0.240
Endothelial vessels	-0.258	-1.499	0.155
F	6.462		
Adjusted R^2	0.477		
D-W	1.874		

Supplementary_Table 3. Multiple linear regression analysis between VM, endothelial vessels and the proportions of Habitat 3 voxels.

Habitat 3 voxels	Standardized Coefficients Beta	*t*	*P*
Discrete VM	-0.298	-1.496	0.156
Continuous VM	-0.708	3.519	0.003*
Endothelial vessels	-0.304	-1.714	0.107
F	5.828		
Adjusted R^2	0.446		
D-W	1.432		

Supplementary_ Table 4. Comparison of VM content, endothelial vessels and different habitat voxel proportions in atypical GBM and PCNSL groups.

Parameter	Atypical GBM(n=10)	PCNSL(n=9)	*Z/T*	*P*
Discrete VM				
M（P_{25}，P_{75}）	1.6（1.6, 3.5）	9.1（4.7, 12.5）	2.539	0.002
Continuous VM				
M（P_{25}，P_{75}）	10%（0, 20%）	30%（10%, 50%）	2.263	0.039
Endothelial vessels				
Mean ± SD	24.3±14.7	20.4±6.8	0.744	0.470
Habitat 1				
Mean ± SD	40.27%±13.65%	55.03%±12.99%	2.408	0.028
Habitat 2				
Mean ± SD	51.23%±11.41%	35.13%±8.98%	3.389	0.003
Habitat 3				
M（P_{25}, P_{75}）	8.30%(0, 11.83%)	4.70%(0, 13.00%)	0.124	0.901

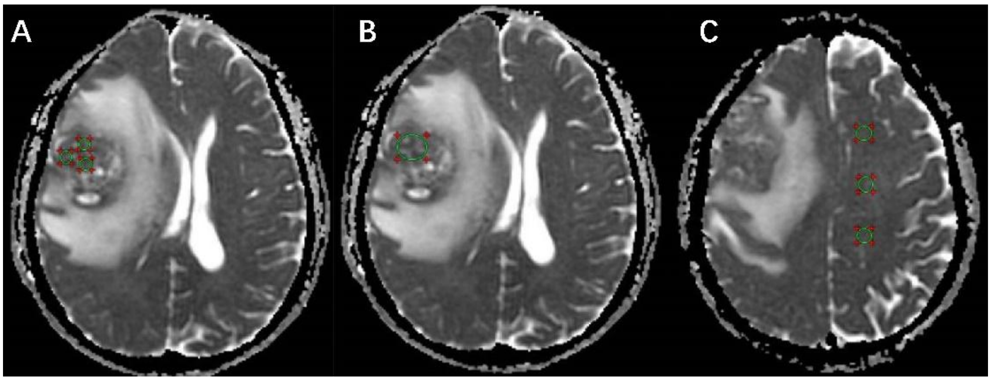

Supplementary_ Fig.1 A: Minimum and maximum ADC measurements. B: Mean ADC measurements. C: Normal-appearing white matter ADC measurements.

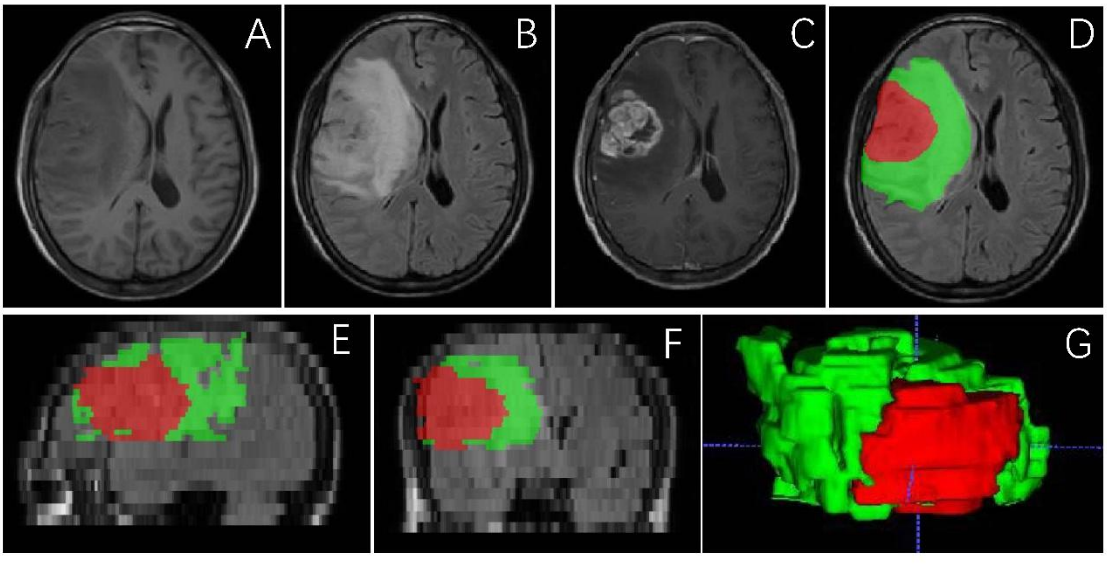

Supplementary_ Fig.2 The voxel count of peritumoral brain edema was measured to assess the extent of edema surrounding the tumor. A: T1WI T1 contrast-enhanced. B: T2-FLAIR. C: T1 contrast-enhanced. D-G: ITK-snap software mapped the ROI of GBM patients with axial, coronal, sagittal, and 3D images (Green is edema voxels, red is tumor voxels).

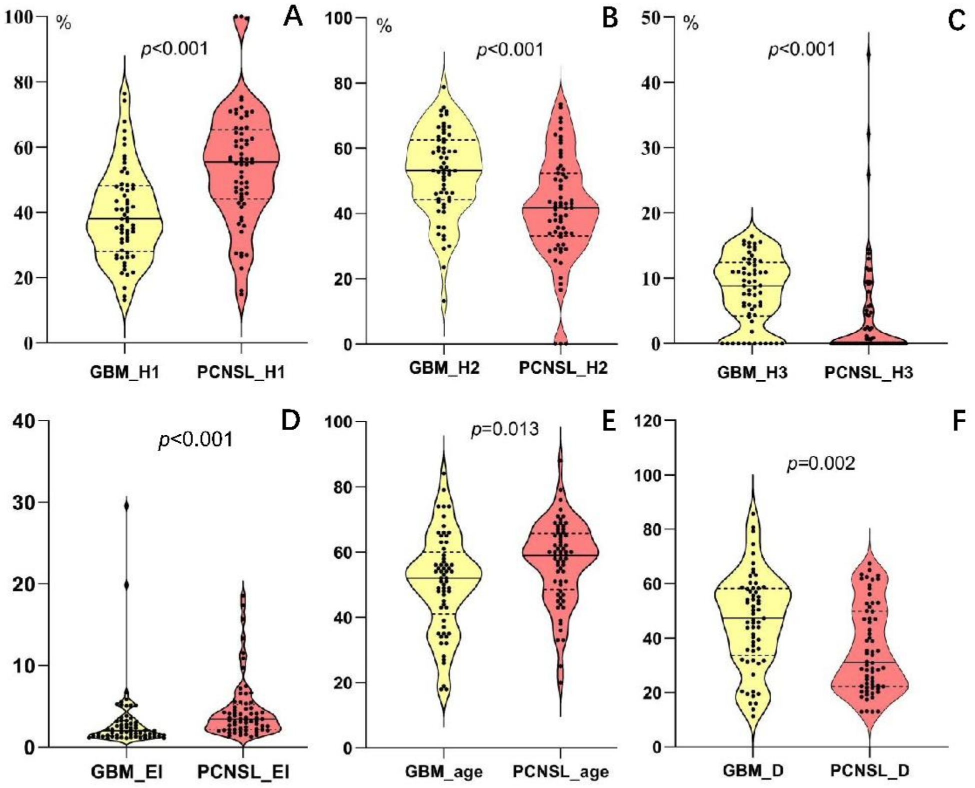

Supplementary_ Fig.3 Violin plot displays the distribution of habitat 1 voxel ratio(A), habitat 2 voxel ratio (B), habitat 3 voxel ratio (C), EI(D), age (E) and tumor diameter (F) in patients with atypical GBM and PCNSL. The solid line indicates the median, the dashed line represents the quartiles. EI: edema index.

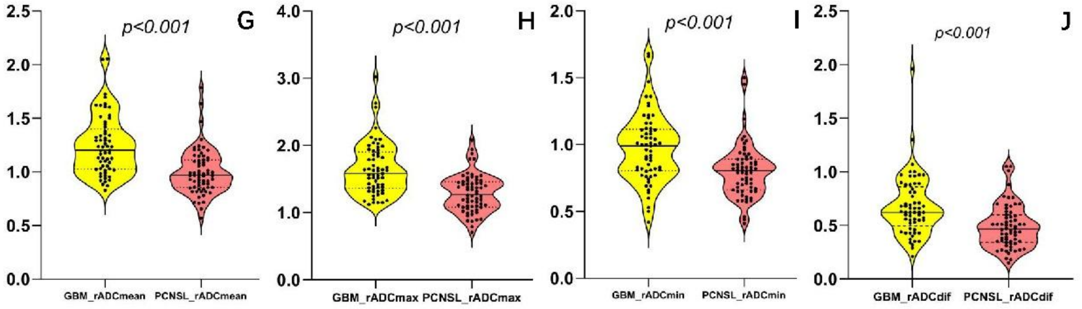

Supplementary_Fig.4 Violin plot displays the distribution of rADCmean (G), rADCmax (H), rADCmin (I) and rADCdif (J) in patients with atypical GBM and PCNSL. The solid line indicates the median, the dashed line represents the quartiles.

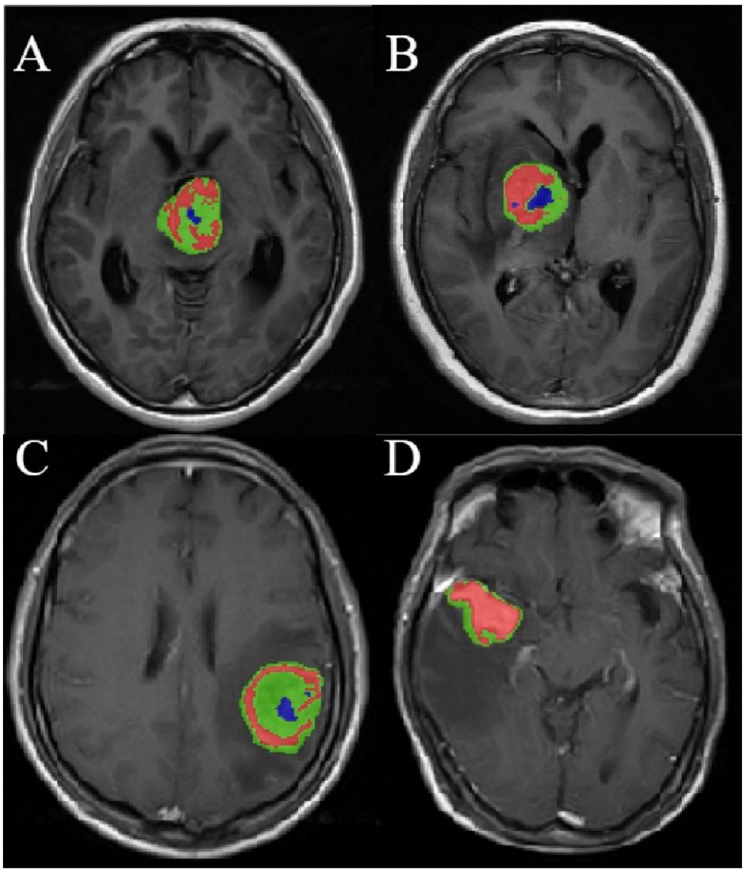

Supplementary_Fig.5 Representative tumor habitat map in patients with atypical GBM and PCNSL patient. A. a 27-year-old female midline GBM patient, the proportion of Habitat 1 voxels is 35.6%, the proportion of Habitat 2 voxels was 62.6%, and habitat 3 occupied 1.8% of the voxels. B. a 51-year-old female midline PCNSL patient, the proportion of Habitat 1 voxels is 44.8%, the proportion of Habitat 2 voxels is 50.5%, and habitat 3 occupied 4.7% of the voxels. C. a 59-year-old male GBM patient with tumor located in the left parietal lobe, where habitat 1 occupied 48.3% of the voxels, habitat 2 occupied 42.8% of the voxels, and habitat 3 occupied 8.9% of the voxels. D. an elderly male PCNSL patient with tumor located in the right temporal lobe, where habitat 1 occupied 70.8% of the voxels, habitat 2 occupied 29.2% of the voxels, and habitat 3 occupies 0%.

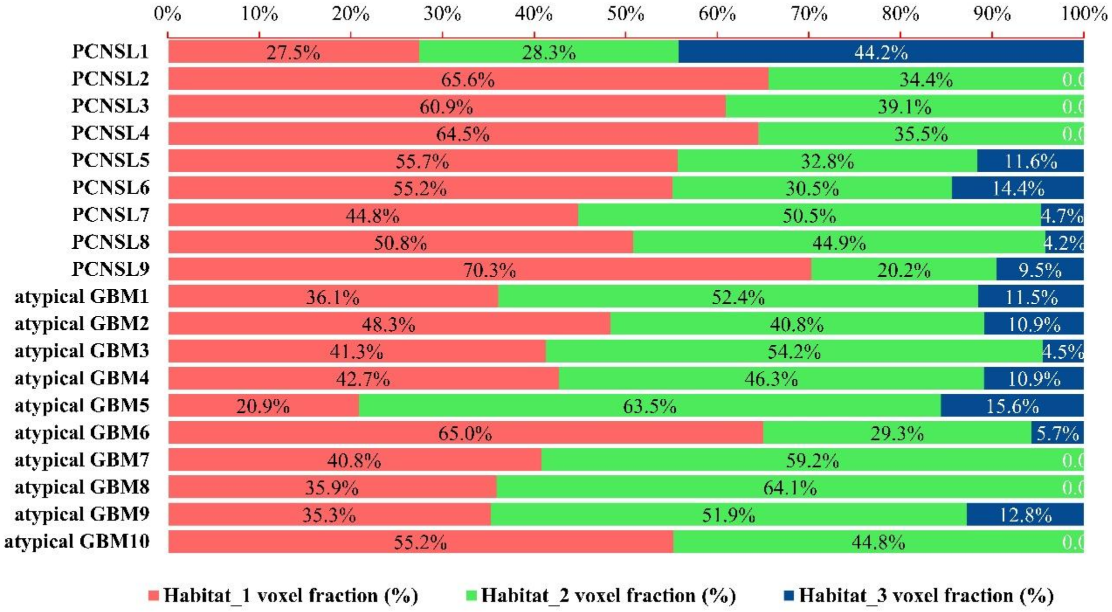

Supplementary_ Fig.6 Distribution of voxel percentage in different tumor habitats in patients with atypical GBM and PCNSL.
